# "The Hospital" Nursing Mirror

**Published:** 1901-07-27

**Authors:** 


### "The Hospital" Nursing Mirror.

Being the Nursing Section of "The Hospital."

[Contributions for this Section of "The Hospital" should be addressed to the Editor, "The Hospital" Nursing Mirror, 28 & 29 Southampton Street Strand, London, W.C.]

### Notes on News from the Nursing World.

### FROM THE "COURT CIRCULAR."

Marlborough House, July 19.—Her Majesty Queen Alexandra received the eighth and ninth thousand Policy Holders of the Royal National Pension Fund for Nurses, including representatives of the Navy, Army, and the Plague Nursing Services, and members of the nursing staff of the principal hospitals and institutions throughout the country. Her Majesty presented certificates to over 500 of the nurses who were qualified to receive them. His Majesty the King and her Royal Highness Princess Victoria were present. The nurses, who had been formed up in the garden of Marlborough House, received their Majesties with a salute by raising the right hand, after which they passed before the Queen, the recipients of certificates being named to her Majesty by Sir Henry Burdett. In attendance upon their Majesties were the Duchess of Buccleuch (Mistress of the Robes), the Lady Alice Stanley, the Hon. Charlotte Knollys, the Lord Churchill, the Lord Colville of Culross, General the Right Hon. Sir Dighton Probyn, Captains Walter Campbell, G. Holford, and F. Ponsonby; the Hon. Sidney Greville, and Colonel Brocklehurst.

### THE PENSION FUND FETE.

The reception by the Queen of the nurses of the Royal National Pension Fund is so fully dealt with elsewhere—our readers will be specially interested in the admirably vivid sketch of the ceremony by an English sister, and the unconventional, warm-hearted account of an Irish nurse, who in sending it says: "I'm afraid this is a bit long, but my heart is too full to limit my lines"—that we need only in this column congratulate the members of the Fund upon the fact that nothing occurred to dim the brilliance of an event which has now passed into history; and the staff of the Fund, from Mr. Louis Dick, the resourceful secretary, downwards, upon the excellent manner in which the arrangements from first to last were carried out. We learn, with pleasure, that many nurses have thoughtfully written to Mr. Dick to express their sense of indebtedness to him for his tireless efforts to promote their comfort and convenience, and we are sure that though it is impossible for him to answer each one individually, they will all understand how thoroughly he appreciates "the shoal of letters" which has reached him, and the kindly spirit which prompted them. A nurse in the Midland counties also suggests to us that the railway companies should receive thanks for their consideration. She says that when the invitation reached her the first thought that occurred to her was whether she could afford the expense, but that that question was answered when she learnt that a return ticket would be issued for single fares.

### NURSING ON THE "AVOCA" AT DELAGOA BAY.

The hospital ship *Avoca* on arrival at Durban received orders to proceed to Delagoa Bay; she remained in anchorage at Durban getting in stores to provide for invalids and staff for about six weeks, and sailed for her work of providing hospital accommodation for the fever-stricken patients collected from Middleburg, Barberton, Crocodile River, and Koomati Poort. The superintendent sister in charge, Miss Makepeace, A.N.S., states that the vessel arrived on Saturday, May 11, and that it was soon found how much a hospital ship was needed. Patients came from shore immediately the anchor was let go. No English doctor is allowed to practise in the Portuguese town of Lourenço Marques. Only persons in Government employ are treated on board, but frequently the doctors are asked by the British Consul-General to see a case. There are no nurses in the town. During the month the *Avoca* has been in the bay 800 patients have been under treatment, a convoy arriving three times a week without fail—generally from about 50 to 70 patients. Each vessel leaving for Durban which had accommodation, took some who went either to Howick, Mooi River, or Pine Town, to recruit before rejoining their regiments, some who were not so debilitated going straight back to Koomati Poort. Even of those considered most convalescent many continued to have repeated attacks of fever, and had to come again within a fortnight, and of course next time they were sent to Durban. In the *Avoca* only new cases could be treated, or the work as a base hospital could not have proceeded. On June 11th the vessel sailed for Durban, carrying about 280 patients. The P. M. O. decided that the very serious cases should not be landed, and about 36 cases—the worst ones—were taken back to Delagoa Bay. So far, there has not been a death, but many have been on the verge, and if the nurses had not been very much on the alert night and day to call the doctor and at once given stimulants, even before he could get there, such a good record of a month's work would have been impossible. Some patients collapsed between the train and the lighter which takes them on board, and brandy was administered at once. A doctor and a sister met each convoy, and the superintendent sister, with one exception, met all twelve. Before the wards were all open she took a sister to help with each convoy, so as to be prepared for any emergency.

### THE THREE YEARS' CERTIFICATE.

We announced the other day that the Sheffield Royal Infirmary is now a three-years' training school, and that no certificate is presented for less than that period of training. We are sure that the authorities will not repent the change they have made. Fresh proof of the wisdom of adopting the three years' period is forthcoming in our issue to-day in the statement of the matron of Derbyshire Royal Infirmary to our Commissioner that, though the abandonment in 1898 of the system of paying probationers with the two years' certificate in favour of non-paying probationers and the three years' certificate, involved a financial loss at the outset of £300 a year, it has not been regretted. Asked whether the candidates for probationership are of the same class under the new, as they were under the former system, the matron said: "Quite as good a class, and we get more suitable women than before." The result of the experiment at Derby should have the effect of encouraging other institutions which still lag behind to boldly put themselves in line with all the great training schools.

### A NURSE ON THE CONCENTRATION CAMPS COMMITTEE.

Whatever doubts may be entertained as to the wisdom of appointing a committee of ladies to visit the Concentration Camps in South Africa, it is a compliment to the nursing profession that the Secretary for War has chosen as one of the members Miss Katherine Brereton, who was in charge of the Yeomanry Hospital, and has rendered valuable service throughout the war. Miss Brereton was trained at Guy's Hospital, becoming sister in 1893. She has also had experience as staff nurse at the Birkenhead Children's Hospital, and the General Lying-in Hospital, whose certificate she holds.

### SIXTY-ONE YEARS A NURSE.

It is announced that Mdlle. Bettard, who has been for sixty-one years a nurse in Paris, is about to retire. She entered the service of the Salpêtrière in 1840 at the age of 18, and will give up charge of her ward on August 1st; but she will not leave the hospital, as under a rule dating from the time of Mazarin, every nurse of more than twenty years' service has the right to board and lodging for the remainder of her life. To Mdlle. Bettard belongs the distinction of having served for the longest period on record.

### NURSING IN BRITISH COLUMBIA.

It will be observed that the nurse in charge of St. Luke's Home, Vancouver, who is at present in London, offers in another column to answer any inquiries that may be addressed to her in respect to nursing in British Columbia. Sister Frances, who confirms the statement that the colony is in need of nurses, thinks that they are particularly wanted in isolated country places, rather than in Vancouver or Victoria. There are two or three points in her interesting letter which merit the careful attention of nurses who contemplate going out to British Columbia. One is that the possession of a certain amount of ready money to fall back upon is required, and another that a knowledge of maternity nursing is indispensable. Her remarks about the nature of the work a nurse in the country districts is expected to undertake should also be digested, and she makes it very evident that none who cannot "rough it" should make the venture.

### THE QUESTION OF UNIFORM AT WALSALL.

The master of Walsall workhouse has been upheld by a narrow majority of the Walsall Board of Guardians in a most unusual and objectionable course. At the last meeting of the Board a letter was read from the probationers in the workhouse infirmary strongly protesting against a proposal of the master to supply uniforms similar to their own to the domestic servants of the house. They stated that when they came to the infirmary to learn their profession they did not expect to be dressed like the cooks and laundresses, and they asked that the latter should be given a different uniform from their own. The master, who was requested to furnish an explanation, said that the house committee gave him discretion as to the uniform the female officials should wear, and "he favoured their having a dress similar to that of the nurses, because he thought that it would tend to allay certain friction which had existed between the workhouse and the infirmary." We are glad to see that several of the guardians, including all the ladies on the Board, desired that the matter should be referred back to the House Committee; but, unfortunately, on a division being taken, the resolution to this effect was lost by ten votes to nine. While the manner in which the master has exercised the discretion conferred upon him proves how unfit he is to possess it, the action of the House Committee in clothing him with such power, and of the majority of the board in confirming their action, cannot be too severely condemned. To assign to domestic servants of the workhouse the same uniform as the nurses of the infirmary is an insult which the probationers have a perfect right to resent, and we accordingly advise the nurses at Walsall to memorialise the Local Government Board on the subject at once. They are quite justified in urging that they would not have entered upon their training if they had imagined that the domestic servants would have been authorised to masquerade in their uniform.

### QUEEN'S NURSES IN SCOTLAND.

At the quarterly meeting of the Scottish Council of Queen Victoria's Jubilee Institute for Nurses, it was stated that they are responsible for eleven Queen's nurses and for thirty-nine probationers at various stages of hospital and obstetric training. During the three months twelve entered for training, nine were on trial, of whom two failed, and eight completed training. Eight nurses received appointments, and nurses under thirty-eight branches were inspected and reported upon to the executive committee. The number of cases nursed in Edinburgh from the training home was 1,353, and the number of visits paid 22,334.

### THEOLOGICAL TESTS.

A curious incident is reported at a meeting of the Derry Board of Guardians. The applications of two candidates for the post of assistant nurse in the workhouse infirmary were considered. One of these had sent in references and her photograph, thus complying with the conditions of the advertisement; while the other had failed to enclose her photograph and had applied through the medical superintendent. The latter applicant, it appeared, is a Roman Catholic, and a lady guardian demanded her appointment on the ground that 72 per cent. of the patients in the infirmary are Romanists. She would have done better if she had been content to urge the claims of the Roman Catholic nurse on the ground that she had enjoyed three years' training, while the other nurse had only received one. This little distinction did not, however, seem to strike the Derry guardians as being at all important, for, though they decided to interview both nurses, they passed it by. We suppose that as individuals they entertain the opinion that it is more necessary a nurse should be a Protestant, or a Roman Catholic, as the case may be, than that she should be fully trained.

### LONDON SCHOOL NURSES' SOCIETY.

The annual meeting of the London School Nurses' Society was held last week, the Countess of Aberdeen presiding. Miss Honor Morten, the honorary secretary, read the report, which stated that the Society had had the full service of three nurses, one half-time nurse, and one voluntary nurse during the past year. The number of schools visited regularly was 63. The Society needed £500 a year if nurses were to be supplied to the poorest schools. Dr. Shuttleworth moved, and Miss Rose Petty, the first nurse appointed by the society, seconded, its adoption, Dr. Shuttleworth observing that there had been many instances of the value of the nurses' work. The society, he pointed out, sent qualified visiting nurses to the poorest schools to attend to the small ailments of scholars, on the principle that there was no more sure way of securing the health of the people than to correct small ills at the beginning. Lady Aberdeen spoke of the difficulties with which the Society, though it had a special claim upon the public because it worked for children, had to encounter—principally, she thought, from the fact of its being but little known; and Lord Shaftesbury, pleading for a cause which his honoured ancestor would have delighted to advocate, said that every shilling subscribed to the Society went directly to the object in view. We trust that the £500 a year needed to keep eight nurses regularly at work in all the poorest schools where they are badly needed, will be speedily forthcoming, and that the committee may not, for the third time be compelled, through lack of funds, to have to consider the question of dissolving such a useful organisation.

### A NURSE'S ROOM ON FIRE.

There were three inaccuracies in the short paragraph, headed "Fire in a Children's Hospital," which appeared in a daily paper this week. In the first place it was stated that the cause of the fire was unknown, whereas, in a chat with the matron, our representative learnt that it was caused by a window-curtain being blown across a lamp by the draught from an open window, just after the home sister had left the room. Secondly, it was said that the fire happened in the morning, whereas it was on Sunday evening that the foreman of the fire-station opposite noticed flames and smoke on the second floor. And thirdly, it could hardly be called an "exciting" fire, except from an outside point of view. "All Shadwell" turned out to see "a fire at the Orspital" it is true, but those inside were not equally perturbed. Of course, the matron said, it might have been very serious if it had not been for the promptitude with which the fire-engine from over the way, and an additional one from Whitechapel, arrived on the spot. She was in her sitting-room, at about a quarter to ten, when there was a sound of strange voices and the tramping of feet, and going into the hall she saw the firemen going upstairs. They did their work quickly and well, and the most serious damage was the destruction of curtains and lamp, the spoiling of the carpet, the blackening of walls and ceiling, and the upsetting of a pail of water over one of the housemaids: this was an accident caused by the anxiety of one of the men employed in the hospital to give all the assistance in his power. The home sister's room, which had recently been distempered, will have to be thoroughly cleaned and redecorated; but all loss is covered by the insurance. One of the nurses, who was in the Commercial Road, was surprised at being accosted by a perfect stranger, who informed her of the occurrence in his own way: "There's a big fire at your place; be quick, or you'll be too late!"

### NURSES AND THE HEAT.

Not only has the recent heat been intensely felt by the Shadwell nurses, but their work has been greatly added to by the usual summer epidemic among the babies. They look for it in August as a rule, but this year it came in July, and the sad thing was that many of the little patients were in too exhausted a condition when they came in for anything to save them, and there have been three or four deaths daily. Sometimes a bed has been filled up twice in one day, so numerous have the applications been, and so frequently has the case proved fatal. But not so many die now as formerly, our representative was told, owing to improved methods of treatment; the trouble is that the babies often do not come until their case is nearly hopeless. The ignorance of parents is appalling. "It's only vomiting and diarrhœa," a mother observed when nurses and doctors were doing everything they could think of to restore her child to consciousness by mustard baths and other means. "What harm can there be in that?" And yet the epidemic was such that in twenty-four hours all might be over and the child dead. The nurses will welcome the cooler weather, especially those on night duty, who have found sleep an impossibility during the day on account of the heat.

### A PATIENT'S GRATITUDE TO A NURSE.

On Wednesday last week Miss Jane Beatrice Williams, who was trained at Croydon Infirmary, and has latterly held the post of deputy matron at the Isolation Hospital, Mitcham, was married at St. Mary's, Beddington, to Mr. Richard Norman Aitkin, of Mitcham. The bride received a large number of presents, two of which she specially valued. One was a marble dining-room clock from the doctor, matron, nurses, maids, and staff of the Mitcham District Isolation Hospital, and the other was a glass *épergne* from a boy for some time a patient under the charge of the bride at the hospital. He was so grateful to Miss Williams for her kindness to him, that on coming out he saved his coppers to get her this wedding gift and was present at the church to witness the ceremony.

### SHORT ITEMS.

Visiting the offices of the Women's Memorial Fund to Queen Victoria in connection with the Queen's Nurses one day last week, the Duchess of Portland, a member of the Executive Committee, was struck with the extra stress thrown upon the staff, especially the women type-writers, by the excessively hot weather, and the following morning the Duke of Portland telegraphed in her name, offering to supply the office with electric fans at his own cost during the continuance of the hot weather.—There have been two more arrivals of sick from South Africa, and the following Nursing Sisters were in charge of the patients on the s.s. *Aurania*:—A. B. Trew (wishes to retire), A. Garden (leave), A. N. Martin and A. G. Wason (return to South Africa), L. M. Speers (time expired). Sisters M. G. Richardson, A. J. Moffat, M. E. Tippetts, and A. A. Bousfield were invalided. The *City of Vienna* brought three Nursing Sisters: M. F. May, M. S. Clarke, and B. Munn (all on leave).—The sum of £50 has been bequeathed to the Chorlton-on-Medlock Sick Poor and Private Nursing Institution, under the will of the late Mr. J. J. M. Blydt, of Rydal Mount, Withington, Manchester.—Nurse Hooker, who nursed at Ilkley, Yorkshire, during the early part of 1900, is requested in a daily paper to communicate with Mr. Alan S. Martin, solicitor, 12 The Avenue, Blackheath, S.E.—Miss McLaren, the new matron of Seaham Harbour Infirmary, was formerly nurse-in-charge of Inveresk Combination Poorhouse Sick Wards.—Miss M. Phillips, formerly Queen's Nurse at Wisbech, is taking up a similar appointment at Pontypool, Monmouthshire.

### Notes of Surgical Lectures to Nurses.

Delivered at the London Temperance Hospital by Dr. W. J. COLLINS, M.S., B.Sc., D.P.H.Lond., Fellow of London University and of the Royal College of Surgeons. Surgeon to the London Temperance and the Royal Eye Hospitals.

THE membranes or meninges which cover the brain are three, the dura mater which lines the cranium and in places by separation of its fibrous layers forms venous sinuses; the arachnoid, a serous membrane thicker at the base of the brain where it is separated from the third membrane, or pia mater, by a space (the sub-arachnoid space). This space is filled with cerebro-spinal fluid and communicates with the ventricles of the brain and the sheath of the spinal cord. This circumambient fluid protects the central nervous system from shocks after the fashion of a water-bed. It may be in excess, as in chronic hydrocephalus, or may form the contents of a hernial protrusion of the meninges between the skull sutures (meningocele), or in the lumbar spine from imperfect closure of the vertebral column (spina bifida). The third membrane, or pia mater, unlike the other two, dips down into the sulci between the cerebral convolutions and into the interior of the brain, supplying it with blood derived chiefly from the internal carotid arteries. Meningitis may result from injury, from extension of inflammation as from disease of the bones of the ear, from tubercle, &c. Among the symptoms which indicate its presence are headache, high temperature, vomiting, convulsions, piercing cry, emaciation, loss of control of bowel and bladder.

The nervous systems of man are two. 1. The sympathetic, which represents the nervous system found in lower animals, even in those that have no back-bone (Invertebrata), as the worm and the fly, and it is chiefly concerned with regulating the processes of organic life. 2. The cerebro-spinal system, found only in vertebrate animals, and most highly developed in man. In only two animals does the absolute weight of the brain exceed that of man, viz., the elephant and the whale; in no animal is the ratio of the brain-weight to the body-weight greater than it is in the case of man. Two sets of elements compose both systems—(1) fibres, (2) cells. The former are collated into nerves, the latter into nerve centres. Fibres, like electric cables, consist of (1) an outer (nucleated) sheath; (2) the axis cylinder, along which nervous currents run; (3) an intervening medullary sheath or white substance of Schwann. This is absent in the sympathetic system and in the olfactory and auditory nerves. The function of nerves is solely to conduct; currents travel at the rate of 120 feet a second. The conduction may be centrifugal (efferent) or centripetal (afferent). Nerve fibres terminate centrally in nerve cells and peripherally in plexuses, end-bulbs, Pacinian corpuscles, touch corpuscles, etc.

The spinal cord, covered by similar meninges to those of the brain, extends in the infant the whole length of the spinal canal, but growing less rapidly than the spine it finally terminates at the level of the first lumbar vertebra, being in the adult about 18 inches long. It consists of two symmetrical halves, and externally is composed of white matter running in columns and receiving or giving off 31 pairs of spinal nerves, each arising by an anterior (motor) and a posterior (sensory, ganglionic) root. The grey matter rich in nerve cells is situated centrally and is disposed in two columns crescentic on section and placed *dos-a-dos.* Besides conducting nerve currents up and down its fibres the spinal cord is a centre for reflex actions (*e.g.* percussion below knee of flexed and flaccid leg producing knee-jerk) and contains special centres (*e.g.*, for controlling sphincters of bladder and bowel). Afferent (sensory) fibres pass through posterior (ganglionic) roots, crossing almost directly to opposite side of cord and through medulla to opposite side of brain. Efferent (motor) fibres in the anterior roots travel by same side of cord crossing at medulla to opposite side of brain. Thus impulses whose source and objective are the left hand and foot are "represented" on the right side of the brain. The spinal cord may be divided, bruised, compressed, or inflamed as results of injuries or disease, or concussion of the cord and certain forms of degeneration (sclerosis) may follow spinal injuries. Paraplegia or complete symmetrical loss of sensation and motion below a horizontal line results from section of cord; such lesion above the origin of the phrenic nerves causes death from paralysis of diaphragm. Bed-sores, involuntary discharge of excreta, pneumonia, are common in fracture or dislocation of spine involving damage to cord. Tubercular disease of spine (Pott's disease) causing destruction of the bodies of the vertebræ and consequent "angular curvature" may by pressure on the cord occasion paraplegia.

The human brain weighs about 50 ounces, is divided into two symmetrical convoluted hemispheres connected by a commissure, and continuous with the spinal cord through the medulla oblogata. To the latter and to the cerebral hemispheres the cerebellum lying in the occipital fossae is united by peduncles. The surface of a cerebral hemisphere is divided by three fissures (Sylvius, Rolando, parieto-occipital), into five lobes (frontal, parietal, occipital, temporo-sphenoidal, and central. A line drawn from the lower margin of the orbit, through the auditory meatus to the occipital protuberance indicates the base of the brain (Reid's line).

A point ½ inch behind the middle of a line drawn from the root of the nose to the occipital protuberance marks the starting-point of the fissure of Rolando which runs downwards and forwards at an angle of 67°. On either side of this fissure the grey matter of the brain surface contains "centres" for movements of the trunk, legs, arms, face, head, and eyes. On the left side the posterior and inferior portion of the frontal lobe is the "centre for speech" (Broca). By the loss of function indicating implication of one or other of these centres injuries and diseases of the brain may be "localised." Paralysis of cerebral origin, by virtue of the crossing or decussation of nerve fibres proceeding to and from the trunk, is usually one-sided (hemiplegia); right hemiplegia is sometimes accompanied by loss of the power of speech (aphasia). Sudden loss of consciousness arising from injury or disease of the brain (often due to cerebral hæmorrhage) is called apoplexy (stroke). A condition in which the brain functions are not abolished but deranged, causing incoherence of thought and violence of action, is delirium: it may arise from poisoning or from injury and subsequent inflammation of the brain or from organic disease. A special form of delirium arising from excessive use of alcohol often complicates surgical cases of injury or operation (delirium tremens). It is accompanied by sleeplessness, refusal of food, busy delusions, loathsome illusions, and tremor. Food, sleep, and abstinence from alcohol are the chief elements in its successful treatment.

### To Nurses.

WE invite contributions from any of our readers, and shall be glad to pay for "Notes on News from the Nursing World," or for articles describing nursing experiences, or dealing with any nursing question from an original point of view. The minimum payment for contributions is 5s., but we welcome interesting contributions of a column, or a page, in length. It may be added that notices of entertainments, presentations, and deaths are not paid for, but, of course, we are always glad to receive them. All rejected manuscripts are returned in due course, and all payments for manuscripts used are made as early as possible after the beginning of each quarter.

### Reception by the Queen of the Nurses of the Royal National Pension Fund.

(BY OUR SPECIAL CORRESPONDENT.)

THE fifth reception of the members of the Royal National Pension Fund for Nurses by the President at Marlborough House took place under the most pleasant conditions. In one respect the ceremony was without precedent, for while on former occasions the hostess was the Princess of Wales, the nurses were on Friday afternoon last the guests of the Queen. In 1899 the function was held in the morning, but it was a considerable convenience to nurses travelling from the country that it was deferred this year until a little later in the day, and yet not too late to prevent them from returning home the same evening.

### Outside the Gates at Marlborough House.

As early as one o'clock the arrivals at the garden entrance began, and long before half-past one a large number of nurses had assembled. The crowd of onlookers observed and commented upon their mode of coming. Some came singly or in couples in hansoms, others in growlers, several had chartered brakes, and a certain proportion, apparently inconsiderable, availed themselves of Shanks's pony. Ladies are supposed to be more inclined to altercations with cabbies than men, but the nurses had none. Indeed, there were cabbies who declined to accept any fare at all, and there were fares who recklessly proffered coins twice the necessary size. But, in spite of the heat, everyone was in the best of tempers, and there were no complaints because of the crush; no lamentations on the part of the holders of white tickets that, when half-past one struck and the gates of Marlborough House were promptly opened, they were necessarily reminded that they were not expected until two.

### Inside the Grounds.

Yet it was with an air of exultation that the owners of the coloured tickets trooped into the grounds. Outside, it was all glare and dust; inside, the beautifully kept lawns, the beds radiant with the fairest of summer flowers, the inviting pavilions, the shady trees, the sheltered nooks, suggested the heart of the country rather than the centre of the capital. In one of the sheltered nooks was the office of the Pension Fund, and here the indefatigable secretary, Mr. Louis H. M. Dick, and his courteous assistants gave information and advice to all who sought it; while in another delightful corner was a commodious cloak-room where the nurses got rid of cloaks and bonnets, bags and baskets, and all other impedimenta. Punctually at two the garden gates were opened again, and there streamed into the grounds the second detachment, who, like the first, lost no time in divesting themselves of their encumbrances, and preparing for the preliminary event of the day.

### The Rehearsal.

This was, of course, the drill by sergeants and sergeants-major of the Royal Marine Artillery and the Royal Marine Light Infantry from Portsmouth, under the direction of Major-General Crease, C.B., Colonel Walter Campbell, A.D.C., groom-in-waiting to the King, and Captain Cottingham, of the Royal Marine Artillery. It is no easy matter to get upwards of a thousand novices to conform to military discipline, but General Crease and his assistants spared no effort to convert the 1,047 nurses into soldiers under orders for the time being, and their success was remarkable. The large cards which had been placed on poles to mark the position of each company of a hundred nurses, and to indicate the numbers which corresponded with those marked on the card held by each nurse, facilitated matters greatly; but it is due to the nurses to say that they paid the utmost attention to the directions given them by the sergeant in charge of each company, and to the Pension Fund Staff to mention that all the details of each individual nurse were worked out at the office the night before the ceremony. The experimental march of the companies was a singularly picturesque feature of the proceedings, and was evidently much enjoyed by those who took part in it. Some nurses were more alert than others to respond to the word of command, but as a body they gave little trouble. There was one who had to be content to watch the drilling from a chair under the trees. She was on crutches owing to a fall three weeks previously, but she was not to be prevented from receiving her certificate from the Queen by such a trifle as a broken leg. It was a real deprivation to her that she could not be included in the rehearsal, but she had her compensation afterwards.

### The Uniforms.

As the nurses, four abreast and arm-in-arm to keep the line, went through the ordeal of drilling, the contrast between their uniforms, their height, and their ages was very noticeable. Blue of various shades was the predominating colour, though the scarlet capes of the members of the Army Nursing Service and the Army Nursing Service Reserve were most conspicuous. But there were red uniforms and green, white, and black; stripes of all sorts, caps of all shapes, aprons of all sizes. Many nurses wore gloves, while here and there, as if to accentuate the nature of their avocation, might be seen the scissors and chatelaine. The Royal Red Cross and the plague medal were prominent among decorations, but medals and badges, either representing rewards of merit or association with some particular institution, were strongly in evidence. There was one badge worn on the left arm by all, namely, the charming red armlet of the Pension Fund; which, owing to the mourning, was partially covered by a band of crape, provided to each nurse before the commencement of the drill. The average height was medium, but there were several little nurses, and there was one at least of remarkable stature. Youth and beauty were strongly represented, but beauty was far from being limited to the possessors of youthful faces, and among the most attractive personalities were nurses whose silvering hair was a crown of glory.

### The Other Spectators.

Towards three o'clock the few guests who had been invited to be present, because of their connection with the Royal National Pension Fund, or the Junius S. Morgan Benevolent Fund, all of course, in mourning, began to put in an appearance, the band of the Coldstream Guards playing during the interval which necessarily elapsed between the completion of the rehearsal and the presentation of the certificates. Among these were Lord and Lady Rothschild, the Earl of Aberdeen, Mr. E. A. Hambro (Chairman of the Council) and Mrs. Hambro, Sir

Henry Burdett, K.C.B. (deputy-chairman of the Council) and Lady Burdett, and the Misses Burdett, Sir William and Lady Broadbent, Mr. C. Eric Hambro, M.P., Mr. Thomas Bryant, Dr. S. H. and Mrs. Habershon, Mrs. Burns and Mr. Walter Burns, Mr. and Mrs. Edward Rawlings, Mr. Alfred de Rothschild, Mr. C. W. and the Hon. Mrs. Trotter, Mr. and Mrs. Falconer L. Wallace, Miss R. Pritchard, Mrs. Farmer, Mrs. Louis H. M. Dick, Miss L. M. Gordon, Miss Katharine Monk, Miss S. A. Swift, and Miss Mary Shirley. These ladies and gentlemen, with the exception of Sir Henry Burdett, remained on the left of the conservatory, outside which the striking Oriental canopy used by the King in India, supported by its silver poles, had been erected to afford a shelter for the Queen.

### The Ceremony.

Meanwhile, the nurses had been drawn up in their companies on the lawn in front of the house, and awaited the advent of their Royal President. Her Majesty, accompanied by the King, the Princess Victoria, the Princes Edward and Albert and Princess Victoria of Cornwall and York, took up her position under the canopy at half-past three to the minute. In attendance upon their Majesties were the Duchess of Buccleuch, Lady Alice Stanley, the Hon. Charlotte Knollys, Lord Churchill, Lord Colville of Culross, General the Right Hon. Sir Dighton Probyn, Captains Walter Campbell, G. Holford, and F. Ponsonby, the Hon. Sidney Greville, and Colonel Brocklehurst. Their Majesties were saluted by the nurses, who raised their right hand, and the band played the National Anthem. Then Sir Henry Burdett, standing to the right of the Queen, proceeded, at her Majesty's request, to present to her by name each of the war and plague nurses, with a summary of her services.

### The War and Plague Nurses.

The nurses thus honoured were twenty-three in number, and would have been twenty-four if Miss Skillman (Sister Hope of St. Bartholomew's Hospital), who was attached to No. 6 General Hospital from January 1900 to 1901, and came back as nurse to Miss Roberts, daughter of the Commander-in-Chief, had not, at the last moment, telegraphed that illness compelled her absence. The nurses actually presented were:—Miss E. A. Dowse, Superintendent Sister of the Army Nursing Service, who organised the nursing service at Ladysmith, was all through the siege, and has been practically under fire the whole of her service; Miss Mary Grenfell Hill, of the Army Nursing Service, who also went through the siege of Ladysmith, and was in the train at Ladysmith Station which was struck by a shell and partially destroyed; Miss E. A. Wilson, A.N.S.R., who was superintendent sister of the Langman Field Hospital in Howitzer Camp, Pretoria, and was also attached to No. 9 General Hospital, Bloemfontein; Miss R. Fergusson, A.N.S.R., and Miss Anna D. Hook, A.N.S.R., who were attached to No. 9 General Hospital, Bloemfontein; Miss Edith Coutts, A.N.S.R., who was attached to No. 8 General Hospital, Bloemfontein; Miss Mary Frances Lightfoot, Miss F. Gertrude Digwood, Miss A. Nixon, A.N.S.R., Miss Beatrice Jones, A.N.S.R., Miss Adeline A. Cable, A.N.S.R., Miss Seabrooke Cooper, and Miss Margaret Penrose, A.N.S.R., who served for different periods at the Imperial Yeomanry Hospital; Miss Mary Everett, A.N.S.R., who was for thirteen months at No. 1 General Hospital, Wynberg; Miss Winifride Kendall, A.N.S.R., who was for three months at Bloemfontein, two at Pretoria, and five at Wynberg; Miss E. M. Baukes, A.N.S.R., who was with the Ladysmith and Transport Service; Miss G. E. Morgan, A.N.S.R., who was with the Natal Field Force, and was invalided home; Miss Caroline Walker, A.N.S.R., who was in Harrismith Stationary Hospital, and was invalided home for enteric fever, arriving in England on the 29th of last month; Miss Marcia Barker, R.R.C., who has recently returned from service in India; Miss Sarah Clarke, R.R.C., who served for twelve months with the West African Frontier Force; Miss S. E. Barker and Miss M. E. Mead, who have been doing plague duty at Hong Kong; and Miss Rose L. Massey, who has been doing plague duty at Bombay.

### The March Past.

When the first list was exhausted, and the nurses on active service had made their reverence, receiving individually a gracious smile from the Queen as Sir Henry Burdett recited their record, the representatives of the earlier enrolled thousands of the Fund passed by in single file. Finally came the 546 nurses who were to have the privilege of receiving their certificates from the President and to each of these the Queen handed the coveted warrant with Mr. Arthur Hacker's singularly appropriate design of the Angel of Mercy. The march past lasted precisely forty minutes, and during the whole of the period the Queen stood, apparently unaffected by the heat. To every nurse she bowed and smiled, but to one, the brave nurse on crutches, she spoke a few kindly words which must have more than repaid the recipient for all the discomfort she had endured. It was amusing to watch the variety of curtseys which obtained the Royal recognition. Some were very orthodox, and were rendered with as much grace as if the nurses had been habituées of Court, but in several instances a friendly nod, or a queer little bob, did duty. The Queen, however, would quite understand that nervousness explained the lack of finish which characterised the obeisance of a portion of her nurses.

### "Too Hot for Speeches."

The distribution having been accomplished without a hitch, and with a rapidity which could not have been achieved without practice, it was anticipated that there would be some speeches. But when Mr. E. A. Hambro, the chairman, had tendered, in well-chosen words, to her Majesty the respectful thanks of the nurses for her kindness in receiving them that day, the Queen replied informally. She acknowledged the thanks, but said that it was "too hot for speeches," and that she "wished the nurses to enjoy themselves" Perhaps, with her observant eye, she had seen that some of the faces of her nurses looked pale, that others seemed tired of standing, and that many used their tickets as impromptu fans. At any rate, her decision afforded obvious pleasure.

### The General's Signal.

Called to attention, as if they had been seasoned soldiers, by General Crease, his order to "dismiss" was promptly obeyed by the nurses, and in a minute or two they were scattered in all parts of the grounds, though not by any means as sheep without a shepherd. For every provision had been made by the president for her guests, and in the refreshment tents the difficulty of some was to decide in what form to take advantage of the hospitality of the King and Queen. But for most the inducements offered by the seductive ice, or the hock cup of the Royal buffets did not prevail; and one of the prettiest spectacles of the day was that of a number of nurses partaking of tea and fruit as they sat upon the lawn and listened to each other's chatter or the music of the band. The Queen and Princess Victoria, with the Royal children, remained in the grounds until most of the company had left, and many of her Majesty's nurses had the privilege of being addressed by their Royal hostess. When, at length, the end came, and the fitful departure of the few who had a journey to perform was followed by the exodus of the many who stayed as long as they could, it was the universal conclusion that the reception by the Queen of her nurses had afforded the maximum of gratification all round—that nothing had happened to mar the enjoyment of a ceremony which comes only once in the lifetime of even a fortunate nurse of the Pension Fund, unless she has tended wounded soldiers in a distant country, or ministered to the victims of the most appalling of diseases.

### Nurses at Marlborough House.

BY AN ENGLISH SISTER.

LONDONERS are quite accustomed to nurses and to seeing them in many styles of outdoor costume, but even Londoners were a little surprised and possibly amused when last Friday, coming from all parts, were to be seen nurses in spotless white aprons and caps; at least, so it seemed to my companions and myself who were among the number invited, as we wended our way westward to the gates of the Royal residence.

How anxiously we had watched the weather, and never did the most elaborate garden-party frocks of latest fashion occupy the minds of the wearers more than did our plain print gowns and linen aprons. Weather, proverbially "Queen's," favoured us, however—there was not even wind to make our hair untidy, or displace our caps, and the brilliant sunshine of an extremely hot day made even city-washed aprons look dazzlingly white.

As we came nearer to our destination we saw from almost every omnibus or cab nurses alighting in costumes of every known uniform, while here and there were dresses that somewhat astonished those who had understood that "Nursing Uniform" was "obligatory." However, the scene was a very novel one, and the nurses looked fresh and happy at the thought of the honour awaiting them.

When the gates were opened and the wide lawns and shady trees of the beautiful garden were seen, we all found it a great relief after the heat and glare of the pavement we left outside.

We were directed to walk by a certain path to the other end of the garden and when we were about halfway round each one was given a narrow crape band with a request that she would put it on above the red and white armlet worn by all members of the Pension Fund. There were a few nurses who had remembered to wear a crape band, and many were the expressions of regret on the part of others, that they had not done likewise. As each one took the sombre band and fastened her companion's for her, a little hush fell on the merriest of them, as a moment's remembrance was given to the memory of the late dear Queen—one who had done so much by her sympathy and interest to help in the ennobling of the nursing profession, and whose example has been so closely followed by members of her family.

Soon after our arrival in the gardens we were put through a form of drill. A measure of anxiety was felt by us all, until we had found our respective places, and we had grasped the directions so elaborately given. Great care and attention were bestowed by the marshals on the organisation, and most of the nurses seemed to get a good deal of amusement out of the whole proceeding.

There was a puzzled hesitation on the part of many when on being shown how to salute Her Majesty, they were addressed as "Queen Alexandra's" nurses. It was an unaccustomed title for members of the R.N.P.F., who have so long known their gracious President as Princess of Wales, and who have proudly called themselves "Princess's Nurses." A moment's thought, however, gave an added desire to welcome right royally our Queen.

Among the nurses were about twenty-five who had recently returned from "Active Service" or "Plague duty," and to them the place of honour immediately facing the canopy under which the Royal party was to stand had been given. Most of them wore the dress of the A.N.S. or A.N.S.R. with the bright scarlet capes that looked quaint and picturesque even amongst the great variety of uniforms to be seen around.

The large company of nurses were arranged in a wide semicircle, and stood anxiously waiting till the Queen appeared. When the time at length arrived the signal was given, and as their Majesties appeared under the canopy, punctual to the minute, all hands were raised to greet them, and many must have wished that they had been equal to raising a cheer to accompany the salute.

Very sweet and gracious our President looked as she bowed and smiled in acknowledgment, and the King most genial as he stood beside her. We were all delighted to see little Prince Edward with Princess Victoria, the white sailor suit of the bonny little lad giving relief to the mourning dress worn by all the Royal group.

The Queen first received, one by one, the nurses who formed the little centre group. Then followed the march past, the nurses walking four abreast, mustering many hundreds. After this, all those who were to receive certificates passed one by one before the Queen, the name being called as she came near. Very interesting we nurses found it, watching the others who passed by after or before our own turn came, and very much we admired the patience and unwearied kindness of Her Majesty, who would not allow herself to show the least sign of fatigue, and we felt quite sorry for the dear little Prince who stood near the whole time, proving surely what inherited training could do, for no ordinary boy of his age would have been equal to the occasion.

The presentation ended, Her Majesty intimated that she did not wish for any speeches, but desired the nurses to disperse and enjoy themselves. She came amongst us and spoke kindly to some, those who were so fortunate prizing the honour greatly. On the lawn the whole time the younger children of the Duke and Duchess of Cornwall and York—such healthy-looking sweet children—had been playing with their nurses, and they were greatly admired. Looking at them one's thoughts travelled on to the parents so far away, and one realised what a sacrifice it must have been to leave them for so long.

Ample provision had been made for tea, and very refreshing indeed we all found it after standing so long. Delicious fruit and ices, and every description of cake and dainty fare was there, and the nurses, who sat in little groups on the lawn, enjoyed it all very much as they chatted with friends whom they had met. Many were the greetings from friends of long ago, fellow workers who had been inevitably separated, and most of us will retain happy memories of that pleasant time of reunion, and gratitude to the beloved President, who, notwithstanding the greater demands upon her time which her new honours must inevitably bring, devoted so long a period to the nurses whose pride it is to bear her name.

### BY AN IRISH NURSE.

At certain times of the year one in the past has been rather apt to look upon the Royal National Pension Fund for Nurses as a painful means for the extraction of hard-earned money. On quarter-day specially it has often been much abused; but the splendid reception on Friday last at Marlborough House given by their Majesties the King and Queen to many of the members of this fund, has for ever obliterated from our minds all sordid sentiments and filled us with an intense desire to induce all our friends to join, too, in the hopes that in the future they may have an opportunity of having a day as full of enjoyment and rest as fell to our lot.

The day was really prepared for us, the drive, three in a hansom, through the streets in our indoor uniform; the wearing of our badge; the sight of so many other nurses in so many different costumes; the good-tempered crowd outside Marlborough House, all tended to prepare us for what was coming. We had to wait until 2 P.M., but found a shady corner and some old friends, so the time passed pleasantly. At last the wished-for hour came, the doors were opened, and we were walking into the garden. Some nurses had brought umbrellas, others sunshades, but we, obeying literally the letter of the law, had come just as we work, so you can imagine how thankful we were to see all those grand old trees. Gloves had been rather a puzzle, but we solved it by arguing that the only gloves we wore in indoor uniform were housemaids' gloves to clean taps and door-handles with, so we went without. We felt at times rather like poor relations when now and then one of our old nurses came across our path in elegant white kids. But what are gloves? We had come to have a real good time, and we cared not what the others thought; we were happy.

Then we went to look for our places. There was no difficulty, everyone was so ready to help, and soon we were chatting away to our companions in the march as if we had known them all our lives. There was no stiffness, no icy formality, nothing but a sense of feeling quite at ease. Everything that could be done was being done for our comfort, and the full consciousness of this just filled our hearts with joy. The band played softly and the time seemed to fly. High up in one of the windows of that royal residence was the little Prince Albert, laughing and waving welcomes to us, such a natural-looking little babe. Later on he was brought out in the grounds in his perambulator, where he just held on to the sides and swung himself backward and forward, and was the very picture of happiness. The little Princess Victoria and Prince Edward eyed us amicably, but it was Prince Albert who made us feel most at home.

Now the band began to play the National Anthem, and we were soon moving arm in arm four abreast towards our beloved Queen. There was no feeling of fear whatever as one by one we passed slowly before her. Never did she seem to relax her vigilance. Each one of all that vast number felt that the Queen had seen her and bowed to her quite apart from all the others. It was a sight never, never to be forgotten.

Then we were disbanded, rejoined our friends, and made our way to the refreshment tables. The *menu* was:—

Sandwiches de Bœuf. Sandwiches de Jambon.
Sandwiches de Volailles. Sandwiches de Langue.

Gelées Macédoines de Fruits au Champagne.

Bavarois au Chocolat. Bavarois à la Vanille.

Compôtes de Fruits à la Bordelaise.

Pâtisseries Assorties. Plum Cakes.

Glaces de Fraises à l'Eau. Café Glacé.

Limonade, Fruits variés.

Getting the good things was a bit of a scramble, but there was no lack. Delicious tea and cream—cream galore. These I think we enjoyed the most; for though the other drinks were all delicious, what does a nurse care for more than a good cup of tea with cream? All the servants were so kind and seemed to enjoy waiting on us. Only too soon came the order to go; we were loth to move and would just have given anything to send up for the Queen a right royal cheer. Our hearts gave it, though there was no outward sign nor sound. We do pray God to bless our King and Queen, and all the Royal family. May they be spared long to give many more of their subjects days of joy as they gave us.

One nurse said to me as we left: "Well, I was always doubtful whether I had really followed my right vocation or not; nursing has been hard, and looking back there have been many thorns in the path, but the joy of Friday has shut off all the past. I am glad now I've stuck to the last."

### The Nurses of Derbyshire Royal Infirmary.

By Our Commissioner.

### A CHAT WITH THE MATRON.

The Nurses' Home at the Derbyshire Royal Infirmary bears a close resemblance to a charming country seat. Standing right away from the infirmary on high ground, with a large lawn shaded by trees, it has a most attractive appearance. Nor is the interior in the least disappointing. There are cosy and comfortable sitting-rooms, one of them containing a piano on the ground floor in front, and at the back are the sisters' bedrooms, those of the remainder of the nursing staff being on the floors above—about fifty in all. Each nurse has a separate bedroom of excellent size with a fireplace, tastefully furnished. There is ample bath-room and lavatory accommodation. Miss Wilkinson, the matron of the infirmary, who conducted me over the home, mentioned that one of the sitting-rooms belongs to the sisters, another to the staff nurses, and the third to the probationers.

"And as to meals?" I inquired.

"The sisters have tea in the home, but with that exception," replied the matron, "the meals are all taken in the dining-room of the infirmary. We find that the most convenient arrangement. But it is a great advantage for the nurses to be out of the hospital when they are off duty."

"How long has the home been built?"

"Seven years. The assistant matron is responsible for the order of the home."

The matron also showed me over several of the wards of the infirmary, which occupies a splendid site in the main road, and consists of a series of plain but well-built blocks. The electric light has been installed for some time in the buildings, and, generally speaking, the patients appear to be provided for on up-to-date lines. The matron's own quarters are on the first floor. Here, resuming our conversation, I asked for information as to the results of the system of three years' training.

"It has not been in operation four years yet, but we are feeling the benefit."

### The Old System.

"Previously," continued the matron, "certificates were given for two years' training, and each probationer paid a fee of 25 guineas. She received no salary, but only her board, lodging, and washing."

"Then, when you started the three years' system, you ceased to have paying probationers?"

"Yes. There had for some time been a difficulty in getting probationers. The premium was heavy, and the two years' certificate does not rank highly."

"What was the number of paying probationers?"

"About twenty-two. The change was made at the beginning of 1898. At the outset it involved a loss of an income of £300 a year to the infirmary, but it was made after full consideration, and has not been regretted."

### The Present System.

"Are the candidates for probationerships now of the same class as they were under the former system?"

"Quite as good a class, and we get more suitable women than before. At first applications were not numerous, as it had become widely known that probationers were required to pay a fee, and the impression remained long after the circumstances had altered. It is desired that the probationers should remain in the hospital if there are vacancies for sisters, and they are efficient; but so far, of course, only a few have served their term, though in the year ending last March, I was able to promote two to be sisters. Another who completed her training has gone to the Hertford British Hospital in Paris as charge nurse, and two more have taken up private nursing. Very few of our nurses, however, prefer private nursing."

"How is the existing staff made up?"

"There are twelve sisters, one of whom acts as assistant matron, and another as housekeeper; and thirty-six probationers in their different stages of training. We employ a few staff nurses from outside temporarily, if there is a pressure of work."

"What is the time of trial?"

"Two months, which count in the three years' engagement, as I think that they always should. It is obvious that if the probationers had not acquitted themselves satisfactorily in the two months, they would not be kept on."

"How many applications do you have every year?"

"Nearly two hundred. A great number of the applicants are unsuitable. Many do not care to work for a year for nothing."

"The salary begins in the second year?"

### A Standard of Height.

"Yes, £10 salary is paid the second, over £16 the third year. Others do not come up to the standard of height, which is 5 feet 3½ inches."

"Why do you require the nurses to conform to that standard?"

"Because our bedsteads and all the furniture are so high that little people would find it very awkward to discharge their duties. I have made one or two exceptions, but I think that the regulation is wise and necessary. In the form of application the weight, as well as the height, has to be stated."

"Do you have a personal interview with each applicant?"

"Invariably, so that I may see what the applicants are really like. It is not difficult to judge whether they are intelligent, or whether they are stupid, self-conscious, or diffident."

"What is the limit as to age?"

"From 21 to 32. I prefer to accept applicants from 24 to 25."

A good many who come for two months fall out when the physician examines them at the end of that time. We are most particular as to the question of health."

### The Course of Instruction.

"Will you tell me your procedure in reference to instruction?"

"The first year the probationers have six months in a surgical ward and six months in a medical ward; the second they are on night duty for six months and day duty for six, occasionally doing staff work. In any event they undertake staff work the third year. For a few months in the final year they are on night duty, and sometimes they are engaged in the theatre or the out-patients' department, which is a very large one. Most of the probationers get two or three months' theatre experience in their second and third year's training.

"I suppose there are the usual lectures and examinations?"

"Lectures are given from October to March each year by members of the Hon. Staff, on surgery and anatomy, and in the second from January to March on physiology and medicine. Prizes are awarded to the first and second year probationers who come out top. This year, for the first time, there have been lectures on general subjects, including infectious diseases, medical and surgical nursing, splints and bandages, with one prize of two pounds ten shillings. We propose to arrange a separate course for the third year probationers."

"Do most of the nurses obtain half the number of marks?"

"Considerably the greater number; in fact, few get below half. They all take the utmost interest in the lectures, in spite of the work in the winter being very heavy. One advantage of the training here is that we get an average of from eighty to a hundred typhoid cases during the year, and also a number of diphtheria cases. The diphtheria patients are isolated. It is usual for all the probationers to have experience in these special wards."

"You have also an ophthalmic ward?"

"Yes, that is another feature of the training. There are twenty beds in the ophthalmic ward, and each nurse spends some of her second year there. We have just opened a children's ward. The children were always in the adult wards until last Christmas."

"Some matrons approve that arrangement. What is your opinion, founded on your experience here, and at Leeds, and South Stafford Infirmaries?"

"I think it must be in the interests of the patients that children under eight, who cannot be kept quiet, should be placed in a separate ward."

### The Work in the Wards.

"How many beds are there in the large wards?"

"Twenty-six. In each of these wards there is a sister, a staff nurse, and two probationers during the day, and one nurse during the night. The probationers in their second or third year are called staff nurses; and one of these is left in charge at night. Each probationer takes a side of the ward, so that such work as cleaning and attending to the patients is equally divided."

"What time are the sisters on duty in the morning?"

"Eight; and they remain on until nine. They are off duty for two hours in the day, and they have one long evening free once a week. None of the sisters has night duty. There is a night superintendent who has charge of all the wards. She has two nights and three days off once in two months, and three weeks and a day's holiday. The sisters have a month's holiday, and the probationers fifteen days the first and second year, and twenty-two days the third, as well as a day and a night off every month."

"The night superintendent must be pretty fully occupied?"

"Yes, the night duty is very heavy; and for the last twelve months we have found it necessary to have 'a run-about nurse' to help the superintendent, who makes what use she likes of her. The place is very scattered, though all the distances are traversed under cover."

"Is there any difficulty in the matter of communication?"

"No; because all the wards are connected by telephone with the night superintendent's room, in the front block."

### Uniforms and Recreation.

"What is the rule in regard to uniform?"

"First-year probationers provide their own uniform. In the second and third years indoor uniform is supplied, but it remains the property of the infirmary. Outdoor uniform is optional, and though it is useful for a nurse to put on a cloak and bonnet when she is in a hurry, I do not encourage the wearing of the ward dress out of doors."

"I see that there is an admirable tennis court. Do the nurses cycle much?"

"Many of them have cycles, and they can easily reach the most beautiful parts of Derbyshire. Bearing in mind the provision made for the nurses and all that there is to be learnt, the authorities thought it reasonable to require that they should serve for a year without salary. This year it is hoped that the new isolation wing, in which erysipelas and septic cases of any kind are to be treated, will be opened, and thus still further opportunities will be given of gaining varied experience."

### In the Relapsing Fever Wards, Arthur Road Hospital, Bombay.

By One of the Nurses.

(*Concluded from page* 210.)

A Pathetic Story.

One of the most pathetic incidents which recurs to me is that of a poor Mohammedan woman, who was admitted with her two starving children during the height of the famine. Her story was the story of hundreds and thousands during that terrible time. She had come from Kathiwar with her husband, sister-in-law, and three children. Now all were dead save these two, would the Madam Sahib save them? We knew that it was impossible to save the younger, the little boy; he was a mere mass of bone, over which the skin was tightly stretched. The drawn mouth, sunken cheeks, hollow eyes, and prominent brow, made a terrible picture of a child. He suffered from chronic diarrhœa, induced by long starvation, and lay day after day, without strength to cry, his little wasted hands restlessly tossing off the bed-covering, or raised above him, helplessly catching at nothing. Bi-bi, the little girl, was not nearly so wasted, and when the fever fell we hoped to pull her through. That hope buoyed up the mother, and she took her son's death very quietly when at last his weary spirit was released. Alas for that fond hope! Suddenly Bi-bi complained of pain in her left side. Then it was found that her heart had become permanently weakened by the fever, and that the slender thread on which her little life hung might snap at any moment. She suffered much, but was wonderfully gentle and patient. When she died the one anxiety of the stricken mother was that the child should be buried by her own caste people. The doctor assured her that this would be done, and kindly promised that she should be told when the body was removed, and so would see for herself that it left the compound under the charge of Musselmans. Never shall we forget the woman's anxiety, as she stood with strained eyes, watching, long before the time to see the last of her darling. At length the bearers appeared carrying the light bier covered, as is the custom of the Mahommedans, with a light bamboo framework, over which was thrown a scarlet cloth. They put it down and waited a few minutes to allow the mother one last look. The convalescent children who were playing in the evening sunshine with their pretty go-cart and wooden horse, left their toys as some one said "There's Bi-bi," and ran to get as near a view as they could. Native children do not dread death. They wonder, but feel no fear, and the Scinde funerals, with their bright-coloured coverings and flowers and tinsel over the bier, with the sweet smell of incense, the beating of tom-toms, the clashing of cymbals and rhythmical chanting of dirges, are, to those of the little ones well enough to take an interest in the outside world, a constant source of delight. Bi-bi's mother stayed with us a little longer; then the Government visitor provided her (out of one of the charitable funds at his disposal) with money enough to return to her native place, where she had still some relations.

Starving Children.

Until last year there had never been any very large number of children at a time in our hospital, but as the distress caused by the famine became intensified they poured in upon us; whilst poor wee mites, left orphans in the plague, smallpox, or cholera wards constantly swelled their numbers; the Relapsing Fever Ward being considered the safest amongst the unsafe places for the bairns—at one time we had over forty of them. Many had suffered much and were past the stage of possible recovery. Some had dysentery in its most acute form, others diarrhœa, sore mouths or horrible sores, and, saddest of all, not a few were stricken with blindness. Plague and cholera are terrible enough, but to our thinking no manner of death is so cruel or so pitiful as that caused by starvation. The slowness, the suffering, and the misery of it, baffle description, and when the victim is a little helpless child the horror is almost unbearable.

The Other Side of the Picture.

But there is a bright side. Many of our little ones recovered, and their quaint ways and merry laughter as they rode their straight-legged horse, or swung to and fro in their roomy swing, were a constant source of amusement and pleasure. How to give them suitable clothing was a matter of some anxiety. Most of the little creatures from Surerath wore nothing save necklaces—two, three, four, or more rows of gaudy beads. Kind friends came to our help and we were soon provided with neat little overalls made by the members of the ladies' guild. With their pretty clothes the children soon put on prettier manners and their improvement was so rapid that, after a few weeks, it was hard to believe they were really the same creatures who had come to us. Then they were semi-savages, terrified at the sight of a white face (this fear is induced by the Indian mothers, who often tell their children that the white man will come and take them away); using their uncut, claw-like nails to scratch the hand that tended them, kicking, biting, screaming, utterly undisciplined and unreasonable.

Food and Cod-Liver Oil.

Feeding time was to them the great event of the day. Two large blankets were spread on the floor round which the children sat. The cooks, with their baskets of rice and buckets of dhàll and vegetables, squatted on their heels in the doorway, whilst ayahs and wardboys had a busy time of it, running to and fro fetching the diets for each. The children would, as a rule, await their turn patiently; but the dear Arab baby always caused a shout of laughter as he dipped his chubby white hand into the rice served to his small neighbour, and scattered it right and left. Feeding time attracted many visitors. The goats used to arrive at the ward doors full half an hour before the cooks, and they would wait without the ring of children popping from time to time their grave faces over the little ones' shoulders as if to remark, "Please don't forget us." Cats, kittens, and pariah dogs all came for a share of the food, whilst the fowls dived in and out, picking up a by no means scanty meal, and the impudent crows flew in and stole as much as they could. After dinner, out came the delightful bottle of cod-liver oil and malt, the contents of which the fortunate recipients regarded as a veritable *bonne bouche*. They used to stand round opening their little mouths like expectant sparrows. It was too expensive a delicacy to be given indiscriminately, and many a longing look was cast at the bottle by those who were strong enough to do without it, while little Kerson, with whom it did not agree, would actually weep for a spoonful. "Mí tūzé páyá pädtó!" ("I fall at your feet!") "Mí tūzé páyá pädtó!" he would reiterate, again and again, accompanying the gesture with the most abject salaams.

A Brother and Sister.

The history of Kerson and his little sister is a very touching one. They came into hospital with their parents, all as ill as they could be. The father died first, but the poor mother lingered for almost three months. All that weary time she lay helpless and usually speechless, save that she would sometimes rouse herself to say a few words to the children who soon became convalescent. The younger child made a good recovery, but Kerson was never well for more than a few days together. His fever would return and his digestive organs had become permanently weakened. At the same time we could never satisfy his craving for food. He had to be taken off full diet again and again. His four-year old sister was devoted to him, and used to secrete for him portions of her own meals, especially the chaupatti, or bhákarí, a native cake made of flour and water, fried in ghee, which is the best-beloved food of the poorer Hindus, and for which they all weary in hospital, from the two-year old baby who can scarcely lisp the word, to the old man or woman who has but a few hours to live. We thought that the children scarcely noticed when their mother died, but it was not so, for soon afterwards Kerson was overheard saying to his sister, "Mottee, we have no one left now. Our father is dead, and our mother is dead. We have only each other. We must take care of each other." Now Kerson, too, has gone to his rest, and merry little Mottee will have to grow up without her brother's love and care.

### Appointments.

Barrow-in-Furness Workhouse Infirmary. — Miss Jessie Cardwell has been appointed superintendent nurse. She was trained at St. Thomas's Hospital, and has since been sister-in-charge of male wards at Bethnal Green Infirmary.

Birkenhead Union Infirmary.—Miss Evans and Miss Barnes have been appointed charge nurses. They were trained at the Birkenhead Union Infirmary.

Bradford Union Hospital.—Miss Ethel Frost has been appointed ward sister. She was trained at the Infirmary, Becket Street, Leeds, and has since been on the nursing staff at the Surgical Home, Spring House, Leeds, and charge nurse at the Workhouse Infirmary, Edinburgh.

Eye and Ear Hospital, Tunbridge Wells.—Mrs. Carew-Hodge has been appointed matron. She was trained at the West Kent Hospital, Maidstone, and has subsequently been sister of four surgical wards at the Royal Sea-Bathing Hospital, Margate; sister of the out-patient department, Hospital for Women, Soho Square, London; holiday sister at Guy's Hospital, where she afterwards remained on as house-keeper; and night superintendent of the Hospital for Consumption, Brompton. Mrs. Carew-Hodge holds a certificate from Queen Charlotte's Hospital for obstetric work, and a certificate for massage and electricity (Weir - Mitchell system).

General Hospital, Dover.—Miss Louisa Fawcett has been appointed sister. She was trained at Kidderminster Infirmary, where she was subsequently staff nurse and sister. She has also been assistant nurse at the Royal Infirmary, Bristol.

Macclesfield General Infirmary. — Miss Adeline Aynsworth has been appointed sister of women's wards, and Miss Nellie Berry sister of children's wards. Miss Aynsworth was trained at Bolton Infirmary, and has since been sister of male and female wards, and night superintendent at the Infirmary and Dispensary, Bolton. Miss Berry was trained at the Queen Victoria Royal Infirmary, Preston.

Northern Convalescent Hospital, Winchmore Hill. —Miss Fanny Dale, Miss Caroline Mary A. Pelley, and Miss Emma Gertrude Hobbs have been appointed charge nurses. Miss Dale was trained at Leeds Union Infirmary, and has since been charge nurse at Kendry Hospital and Keighley Infirmary. She has also been engaged in private nursing. Miss Pelley was trained at St. Bartholomew's Hospital, and has since been on the staff of the British Lying-in Hospital, London. She has also been engaged in private and district work. Miss Hobbs was trained at Guy's Hospital, and has since been on the staff of the Royal South Hants Infirmary, Southampton.

Salford Royal Hospital.—Miss Maud Calvert has been appointed matron. She was trained at Guy's Hospital, and has since been matron at the Town and District Hospital, Newark-on-Trent, matron at the Seamen's Hospital, Royal Albert Docks, London, and matron at the Hospital for Children, Athens.

St. Mary's Infirmary, Highgate Hill.—Miss Edith Hedges has been appointed assistant matron. She was trained at St. Bartholomew's Hospital, and has since been house sister at "Bart's," sister of surgical floor and operating theatre at the Hospital for Women, and matron of the British Hospital, Cannes.

West Derby Union Infirmary, Everton.—Miss Priscilla Harding has been appointed assistant matron. She was trained at Guy's Hospital, where she has since been staff nurse and night superintendent.

### Everybody's Opinion.

### SECTARIANISM IN NURSING.

"Equality" writes: I have just read wieh much interest a letter on "Sectarianism in Nursing." Two cases of "religious intoleration" in a hospital have lately come under my notice, when the converts were "requested to resign" by the "Board!" In this twentieth century such narrow-mindedness seems incredible, but it is unfortunately true. I fancy our Catholic sisters are almost too ready to "suffer in silence." If the matter were brought more prominently before the public, surely the English love of justice would assert itself to place things on a more satisfactory footing.

### THE ACTION AGAINST THE SUNDERLAND NURSING INSTITUTE.

"Mrs. Marriner," matron of the Nursing Institute, Sunderland, writes: My attention has been drawn to the reports respecting Nurse S. H. Foote's action against the committee of the Sunderland Nursing Institute. The report, as it appears, may convey a wrong impression, so I shall be glad if you will allow the following facts to appear in your paper:—On August 30th of last year, Nurse Foote returned to the institute with a swollen face, and I, at once had her seen by Dr. W. H. Maling. He found an abscess forming, and when ready opened it, and saw her several times, and in about a fortnight it was healed. As nurse had never seemed strong, I kept her in for rest until September 27th, she having then been in the institute exactly four weeks, for rest and attention, on that date; I then sent her for three or four days to a lady in the country, who required no real nursing, and to stay with a lady till her own nurse was at liberty, so that she might have change without work. On November 19th nurse asked to go into the town, and on her return informed me that she had been to consult Dr. Squance. I told her that I could not allow any treatment in the institute by an outside doctor without the sanction of our honorary medical attendant, Dr. W. H. Maling. On November 20th nurse went to the lady for whom she was booked, and in the course of two or three days informed me she was much better, and I did not see her again until December 10th, when she came to ask me for three days' holiday, after finishing her case on December 20th. I gave permission, but immediately added that she must have her face attended to. She then explained that that was why she wished the holiday, and that Dr. Squance was going to operate at her patient's house. I then withdrew the leave I had granted, for two reasons, the first and greatest being that it would be simply impossible to work the institute on such conditions; the second reason was that Dr. Squance not being her patient's doctor, there might be professional troubles. As to the statement made in court that owing to the delay in the operation a large piece of the jaw-bone had become diseased, after Dr. Squance had opened the abscess, when syringing a day or so afterwards, a piece of bone came away, which the doctor said was "about half the size of a pea." Nurse Foote could have gone to her home in the South, and under her own doctor, had she wished; but it was not convenient to her. Dr. Squance was not her own doctor: she had simply been sent by me to nurse patients of his, and it was in this way she became acquainted with him as a medical man. I have received a letter from the committee saying that they "sympathise with the matron of the institute, Mrs. Marriner, and the honorary medical officer, Dr. W. H. Maling, in the annoyance which the recent action by Nurse Foote has caused them, and beg to express their continued confidence in them, and their thanks to them for the assistance which they have given in the course of the action."

[Our report was but an abstract of published reports which we printed without comment.—Ed. "Hospital."]

### NURSING IN BRITISH COLUMBIA.

"Sister Frances" (in charge of St. Luke's Home, Vancouver) writes from 26 Trebovir Road, Earl's Court, S.W.: I have just reached London from Vancouver,

British Columbia, and I read with surprise the information in THE HOSPITAL that half a score of nurses are needed in Victoria, or rather British Columbia. I think I am the oldest established nurse out there and can speak very definitely on the subject, so perhaps some of your readers, and especially those who contemplate nursing there, may be glad to hear about it. Victoria has a good hospital and training school well established, and these nurses will, of course, come first with doctors and patients till the new arrival has made her name—I mean taken a case or two and given satisfaction to the doctor, the patient, and the friends. Victoria itself is not large enough to support an influx of strangers, though the surrounding country might; but I fear that "THE HOSPITAL" NURSING MIRROR would be the recipient of many interesting letters about the hardships and makeshifts if many nurses go out. It is when we are sent into the country that our troubles begin, if we can call them so; but it is in this direction that the Canadian nurse shows her adaptability. English nurses especially will not adapt themselves to new countries and people's ways. Don't please come down on me for so sweeping an assertion, but this, as yet, has been our experience with those previously settling among us. In Vancouver we have hospitals and good training schools, plenty of private nurses, and always St. Luke's Home to fall back upon to fill in vacancies, and I do not think that there is an immediate need there. One or two could doubtless get well established, but I could mention many instances of good English and colonial nurses coming out in a few months, perhaps only weeks, applying to me asking what I could advise, and in one or two instances offering to come to me for board only. I help all I can, provided that nothing has transpired to their detriment. I know an instances of a first-class nurse with every necessary certificate, who was glad to come for three months' residence, and so get a good start as the introduction to medical men. Another first-class nurse, equal in all respects to anyone out here, took rooms, waited for work, and at last reached me without one penny. Both are now doing well, but out of a batch of new nurses arriving probably half would not have adequate means or sufficient friends to keep them till they are assured and have a standing, and how will they live? All need money to start with—not shillings, but pounds. Also all who go out must know maternity nursing. This is the only constant branch which always pays, but it means hard work too. In the country districts the nurse takes the mother's place, doing housework as well as nursing. Washing for child and mother, also, is always expected. The fees are 15 dollars a week—not very much more than a good English nurse could get. British Columbia itself sadly needs nurses, but in the isolated country places. Many of the nurses we have are missionary nurses and do not mind what they do, being thankful if they fall into comfort and luxury, but not minding poverty and hardship. Besides a little capital, anyone emigrating must have good health, and not be afraid of rain, for we have plenty; and all must be able to do cooking and housekeeping. I do not want to say that Vancouver and Victoria expect all nurses to engage in domestic work, but in the country places it is absolutely needful as well as in many places in the town, for everyone is not rich, and the servants are Chinese and Japanese, so that there are many things a manservant cannot be asked to do. English nurses, as yet, are not on the whole a success in British Columbia, as the right sort have not come out. Canadian girls can all turn and do round a house, or go into the country and live isolated, and drive or boat as occasion demands, and it grates on people's ears when a nurse who is employed and well paid, her doctor feeling dependent upon her, to hear her complaining because she has had to wash her own clothes, or found the patient downstairs in a stuffy room and had to scrub or clean out another so as to improve matters. This is what a nurse is expected to do and is paid for. Before any nurse goes out she should decide her place, and none should rush off to Victoria or to Vancouver unless she has a definite letter and invitation from a good medical man out therre. Each doctor employs his own nurses, so that a nurse often only works with one for a long time. I shall be glad, if anyone wants any particular information, to answer any letter, but I could not give an interview unless first arranged. I am visiting England and return to British Columbia in August."

### The New Issue at Newton Abbot.

THE development of the nursing question at Newton Abbot in connection with the request of Dr. Culross to the Guardians to pass a general resolution deprecating the questioning of patients and nurses as to his diagnoses and treatment of cases, has resolved itself into a personal quarrel between Dr. Ley, the only medical member of the Board, and Dr. Culross, the medical officer. Dr. Culross has informed the Board that he has ready to place before the committee which they have appointed:—(1) Written statements by two nurses with regard to the conversations which took place between them and Dr. Ley with reference to cases in the Infirmary; (2) a statement of the present superintendent nurse; (3) a statement by the late superintendent nurse.

The medical officer at the same time intimates that he personally has no evidence to offer; that he declines to discuss the diagnoses or treatment of any case with the Board or any individual member; and while he asserts that he has no desire to hinder Dr. Ley in any good work he is doing as a guardian, he claims the co-operation of the Board to stop irregular proceedings. Dr. Ley, in a letter to Dr. Culross, says that the charge against him is as incomprehensible as it is untrue, and adds:—"I most certainly have never questioned patients or nurses as to your diagnoses and treatment of cases nor commented adversely thereon. . . . I do not know, nor ever ask, what is the matter with the patients, for I learn what I require from the patients' cards above their heads. . . . Since you have been medical officer I have supported you in every way except on one—the night nurse question. On this I maintained my right as a guardian to express my opinion. I have striven in every way to raise the nursing of the sick poor to a higher level than the disgraceful condition I found it in 1893; to see that there was an adequate number of properly paid nurses, and also to see that the medical officer should be properly salaried. In return I get an unwarranted attack from the very person I have helped more than any official on the Board. . . . I shall insist on the matter being thoroughly thrashed out. That you can have taken the action you have without being deceived by some unscrupulous person I cannot believe, and I will do my best to see that that person, or persons who have been the means of propagating such falsehoods, shall be exposed." Dr. Ley openly expresses his determination to fearlessly exercise his rights as a guardian, and use his influence to obtain for the old, sick and infirm in the Union House the liberty of a home, and not the restraint of a prison.

### Presentations.

THE matron, Miss Penny, who for private reasons is leaving Salford Royal Hospital aftef a service of over 22 years, has been presented by the nurses and servants on her relirement with a silver tea service which bears the following inscription:—"Presented to Miss Penny by the nurses and servants of the Salford Royal Hospital as a token of deep respect. July, 1901.

### Where to Go.

"THE MORALITY PLAY," in the open air at The Charterhouse, Saturday afternoon, July 27th. Tickets only (2s. each) from W. Poel, Esq., 90 College Street, Chelsea, S.W. The proceeds are given to the Queen Victoria Memorial Fund.

### Echoes from the Outside World.

### AN OPEN LETTER TO A HOSPITAL NURSE.

An interesting ceremony took place at Marlborough House on Monday, when the King received a medal and an address from the Committee of American ladies who initiated the work of the hospital ship *Maine* in the South African war. The medal is of gold, and on one side is a representation of the *Maine* with a red cross in enamel below it, while above is an American eagle holding in its talons a scroll with the words "Presented by the American Ladies' Committee." The medal was originally meant for presentation to the late-lamented Queen, and accordingly on the other side is the following inscription: "Presented to H. M. Queen Victoria in commemoration of the work done by the hospital ship *Maine* for the sick and wounded in South Africa and China, 1899 to 1901." The King, in accepting the medal and address, said the fact that the former had been intended for his beloved mother would make it a specially valued possession, and thanked the committee for "the loyal and dutiful sentiments" expressed in their address.

Most of you know all about the League of Mercy and of its connection with the Prince of Wales's Hospital Fund. It has lately celebrated its first birthday, and considering that it is still a child of such tender years it has done exceedingly well, for it has been the means of adding £9,000 to the funds of the various hospitals. Its mission was explained under most pleasant conditions last Thursday, when a garden party was given at The Lodge, Porchester Square, the object of the gathering being to enlist the sympathies of Paddington in the work of the League. Sir Henry Burdett spoke of his desire to put fresh life into Paddington, and to rouse it up to its responsibilities, and though Sir John Aird, the Mayor, naturally deprecated the charge of local lifelessness, he admitted that much could be done by combined effort. The truth of this statement was borne out by the mention of a lady in Paddington who brought in £20 to the fund during the year, which she had collected in her district, mainly in farthings. What one lady can do of course others can accomplish, and district visitors, the heads of large establishments, and employers of labour more especially, could, I am sure, if they took up the work in real earnest, help materially to replenish the often sadly empty coffers of many of the hospitals. It is the shrinking from personal service which stands most in the way. Many who have the means would willingly give their 10s. a year themselves, but they do not like asking twenty people to contribute 1s. a year each. Even those who could not afford to give 1s. at once could give 1d. a month if the members would not grudge the trouble of collecting the pence. But, alas! very few of us care to exert ourselves if we can possibly get things to jog along without.

Naturally, sympathy has been manifested in England, as in other countries, for Mr. Kruger in consequence of his wife's death. Although the old man allowed his selfishness to reign paramount, and he escaped from South Africa, leaving his wife behind to the tender mercies of a conquering foe, yet he must have had, in his strange way, some affection for the woman who had always made his comfort and well-being her first object in life and had been the mother of his seventeen children. Mrs. Kruger was a typical Boer woman, caring little for externals, and leaving the affairs of State entirely in her husband's hands. She was intensely averse to travelling, and it is said that she had never entered a railway train. The last few years of her life were so overshadowed by the loss of so many dear to her, by the desolation of her land, and by the departure of her husband, that her death as a captive in her own capital, guarded by British soldiers, seems only a fitting *finale*. The funeral took place on Sunday, a special service being held in the famous Dopper Church, opposite the old Presidency, before the *cortège* proceeded to the cemetery. It is not expected that Mrs. Kruger's death will modify the situation, though her husband's health is temporarily affected.

The late Miss Eleanor Ormerod was always so averse to publicity that it is quite possible her name may be unknown to some, although as an entomologist she had a world-wide reputation. As lately as last February she wrote, "My work now literally girdles the earth," and mentioned Japan, China, India, Smyrna, most of the countries of Europe, as well as Canada, the United States, South Africa, and Australasia, from all of which sources specimens and inquiries reached her. Her life illustrates once more the truth that good frequently comes out of supposed evil, for owing to constitutional delicacy, which necessitated long periods of out-of-door life with abstinence from ordinary study, she was enabled to give full play to her natural bent for entomology. She was brought up, as it were, amongst the insects and the plants, and learnt from observation many important points which she could never have acquired from books. She came of a talented family, her father being George Ormerod, D.C.L. and F.R.S., and as he advanced in years she took a large share in the management of the farm and the estate, and this gave her practical knowledge of agricultural affairs. For ten years she contributed largely to the collection of insects made by the Royal Horticultural Society, and for this they awarded her the Flora Medal; while later on the Moscow University gave her gold and silver medals for her accurate models of insects. In 1877 she issued a report on injurious insects which was much valued, and she continued to do so every year till her death. Miss Ormerod was the first lady upon whom the honorary degree of Doctor of Laws of Edinburgh was ever conferred, and the first female Fellow of the Meteorological Society.

Earl Russell has been tried in the House of Lords by his peers for felony in marrying a second time whilst his first wife was alive. He has been convicted of bigamy and sentenced to three months' imprisonment as a first-class misdemeanant. Were it not for the extraordinary circumstances under which the trial took place it would not be worth recording, for the first Lady Russell had already obtained a decree *nisi* for a divorce, and the second wife knew the exact facts of the case before she wedded Earl Russell. But the procedure at the House of Lords, where 200 peers all clad in their robes, 11 judges wearing their usual garments of office, their full-bottomed wigs and their academic hoods, had assembled, was so exceptional that it cannot be passed over. Norroy King of Arms, from a place on the steps of the Throne, where there, however, sat no king, directed the order of the procession. First, the judges were bidden to rise in their places, and proceeded to the Royal Gallery, the juniors leading. Then the barons were called, juniors again leading. Next the bishops joined the procession, then viscounts, then earls. These were followed by Lord Salisbury and six marquesses, three dukes, the Lord Privy Seal, and, lastly, the Archbishop of York. The whole business, with the bowing, waving of staffs, and calling of names, etc., reminded one irresistibly of the trial scene in "Alice in Wonderland," though the end was less strange. The expense of the proceedings was relatively enormous, the fitting up of the Royal Gallery alone for the occasion costing nearly £400, and as eleven judges were withdrawn from their regular work for the day, the delay and expense caused to litigants were of course really serious. Earl Russell has taken possession of the rooms in Holloway Gaol which were occupied by Sir John Willoughby after the Raid. He has to rise at 5 A.M., and is supposed to breakfast on cocoa and bread, but as he may have almost any luxuries he likes brought in from outside, he is not likely to keep closely to prison diet. Also he may have the use, I believe, of more comfortable furniture, so that he will avail himself of something less simple in style than the bed, the washstand, the water-jug, the chair, the looking-glass, the cup and saucer, the plate, and the salt-cellar of salt, with which the prison authorities are bound to provide him.

### For Reading to the Sick.

"THE HARDER TASK OF STANDING STILL."

O POWER to do! O baffled will!
O prayer and action! ye are one.
Who may not strive, may yet fulfil
The harder task of standing still,
And good but wished, with God is done!

*Whittier*, "The Waiting."

Here, and here alone,
Is given thee to *suffer* for God's sake.
In other worlds we shall more perfectly
Serve Him and love Him, praise Him, work for Him,
Grow near and nearer Him with all delight;
But then we shall not any more be called
To suffer, which is our appointment here.
Canst thou not suffer then one hour,—or two?
If He should call thee from thy cross to-day,
Saying, It is finished!—that hard cross of thine
From which thou prayest for deliverance,
Thinkest thou not some passion of regret
Would overcome thee? Thou wouldst say, "So soon?
Let me go back, and suffer yet awhile
More patiently;—I have not yet praised God."

*Mrs. Hamilton King*, "The Sermon in the Hospital."

If anywise from morn to morn
I can endure a weary faithfulness,
From minute unto minute calling low
On GOD Who once would answer;—it may be
HE hath a waking for me, and some surprise
Shall from this prison set the prisoner free,
And love from fears, and from the flesh the soul.

*Myers.*

Common sense never yet cured any Slough of Despond that I ever heard of; it takes you over the Hill Difficulty, but, the very essence of the Slough of Despond is, that it is a dissolvent of common sense—a dull sluggish water, an overflow of that stream wherein Dante saw the melancholy souls.

Common sense deals with facts, the Slough of Despond is made of feelings.

You must go higher than common sense if you are to be saved from Despond. In this very matter of health limitations, common sense tells you that every one has their tether—though their neighbours are not always aware of it—and this does not make you much more resigned to your own; but something better than common sense tells you that the tether of health is one of the most bearable, because it comes most straight from God's hand. Here you find a remedy which goes deeper than common sense. He who loves you tells you to keep still to please Him. You can either let your tether be a mere tiresome restriction, or you can let it "tether you as His Lamb there," securing you in green pastures beside still waters.—*Lucy Soulsby.*

Ill that He blesses is our good
And unblessed good is ill,
And all is right that seems most wrong
If it be His sweet will.

*Faber.*

### Notes and Queries.

**The Editor is always willing to answer in this column, without any fee, all reasonable questions, as soon as possible.**

**But the following rules must be carefully observed:—**

**1. Every communication must be accompanied by the name and address of the writer.**

**2. The question must always bear upon nursing, directly or indirectly.**

**If an answer is required by letter a fee of half-a-crown must be enclosed with the note containing the inquiry.**

*Rules.*

(164) Can you recommend a book of rules for the management of district and private nurses. What are the advantages to a country branch of affiliation with the Queen's Jubilee Nurses?—*I.C.*

There are no books of rules for the management of nursing associations, as each association must make rules in accordance with the circumstances of its own requirements. One of the advantages of affiliation with the Queen's Jubilee Nurses would be advice on this subject; another help is the securing a properly trained nurse, and competent supervision at intervals of her work. Write to the General Superintendent, St. Katherine's Precincts, Regent's Park, N.W., for information,

*Dress.*

(165) What is the most suitable dress for a candidate to wear when applying for a vacancy as probationer. Is a coloured dress with sailor hat suitable, or is a black dress with bonnet better? 2. Should she take a medical certificate with her? 3. Kindly tell me where I can get a medical dictionary, and the price.—*F.C.*

The neater and more quiet the dress the better. 2. The medical man appointed by the hospital authorities will examine you if you are accepted as probationer. 3. Miss Honnor Morten's "Nurses' Dictionary of Medical Terms and Nursing Treatment," price 2s., will suit you. Write for it to the Scientific Press.

*Epileptic.*

(166) Would it be possible to get a poor man into the Epileptic Colony? How should I set about it?—*District Nurse.*

You must apply to the Secretary, the National Society for the Employment of Epileptics, 12 Buckingham Street, Strand, W.C., and fully state the particulars of the case.

*Nursing Associations*

(167) I am a trained nurse; will you kindly tell me of any nursing home I could join?—*M. K.*

We cannot recommend particular homes; but if you consult our advertisement columns you ought to be able to find some to suit you.

*Appendicitis.*

(168) I have heard that the cause of the increased number of cases of appendicitis is caused by the use of enamelled-iron ware. Can you tell me if this is true?—*J. F.*

Of course it is not.

*Nurses' Co-operation.*

(169) Will you kindly tell me if there is any nurses' association in the provinces conducted on the same lines as the Nurses' Co-operation in London, and unattached to any private venture?—*M. E. P.*

The Nurses' Co-operation is, so far, the only association managed by nurses solely for the benefit of nurses.

*Rheumatism.*

(170) I am interested in a poor man crippled with rheumatism. I cannot help thinking that proper treatment, now that the warmer weather is here, might restore him sufficiently to enable his resuming work. Where is there a suitable home?—*Clara.*

Write to the Secretary of St. John's Brine Baths Hospital for Poor Patients, Droitwich; to the Devonshire Hospital and Buxton Bath Charity, Buxton; and to the Royal Mineral Water Hospital, Bath, or you might inquire at the Tallerman Institute, Welbeck Street, W.

*Lepers. Plague.*

(171) (1) I have written to the leper hospitals Ottawa, Madras, and Robin Island, but have received no reply. (2) Can you tell me if nurses are sent out to nurse the plague case in Cape Colony?—*Enquirer.*

(1) You might write to the Organising Secretary the Mission to Lepers in India and the East, Exeter Hall, Strand, W.C. (2) Some have been sent out, but there is no present demand for more.

*Massage.*

(172) Can you recommend a good teacher of massage and electrical treatment in Dublin?—*S. G.*

There is no public course of instruction given in Dublin. The Secretary of the Society of Trained Masseuses, 12 Buckingham Street, Strand, might be about to recommend you a reliable private teacher.

*Lady Roberts' Nurses.*

(173) I am anxious to join Lady Roberts' staff of nurses, will you tell me where I may obtain the necessary information?—*M. E. J.*

Lady Roberts' nurses are privately selected, no outside application for vacancies is received.

**Standard Books of Reference.**

"The Nursing Profession: How and Where to Train." 2s. net; post free 2s. 4d.
"Burdett's Official Nursing Directory." 3s. net.; post free, 3s. 4d.
"Burdett's Hospitals and Charities." 5s.
"The Nurses' Dictionary of Medical Terms." 2s.
"Burdett's Series of Nursing Text-Books." 1s. each.
"A Handbook for Nurses." (Illustrated.) 5s.
"Nursing: Its Theory and Practice." New Edition. 3s. 6d.
"Helps in Sickness and to Health." Fifteenth Thousand. 5s.
"The Physiological Feeding of Infants." 1s.
"The Physiological Nursery Chart." 1s.; post free, 1s. 3d.
"Hospital Expenditure: The Commissariat." 2s. 6d.

All these are published by THE SCIENTIFIC PRESS, Ltd., and may be obtained through any bookseller or direct from the publishers, 28 and 29 Southampton Street, London, W.C.

